# Barriers in cancer care for patients with mental illness – a qualitative study

**DOI:** 10.1192/j.eurpsy.2024.437

**Published:** 2024-08-27

**Authors:** A. N. H. Vendelsøe, M. Stie, P. Hjorth, J. Søndergaard, L. H. Jensen

**Affiliations:** ^1^Department of Oncology, Lillebaelt Hospital, Vejle Hospital, Vejle; ^2^Department of Regional Health Research, Faculty of Health Sciences, University of Southern Denmark, Odense; ^3^The Psychiatric Department in Vejle, Mental Health Services in the Region of Southern Denmark, Vejle; ^4^Department of Public Health, University of Southern Denmark, The Research Unit for General Practice, Odense, Denmark

## Abstract

**Introduction:**

Patients with mental illness experience a substantial inequity when facing cancer treatment compared to patients without mental illness. They have a higher cancer mortality and are less likely to be referred for treatment following clinical guidelines. The cancer treatment can exacerbate mental symptoms, which may lead to discontinuation of the treatment. Other relevant specialities such as psychiatry and general practice are rarely involved.

**Objectives:**

In this qualitative case study, the needs, barriers and facilitators of providing high quality, patient-centered care to patients with cancer and pre-existing mental illness were explored. Emphasis was on patients’ experiences of being in the field between oncology, psychiatry, general practice and the municipality.

**Methods:**

The study was anchored at the Department of Oncology, Lillebaelt Hospital, Vejle and data collection took place from January to June 2023. Through purposeful sampling five patients with cancer from adult psychiatric setting were included. Field studies were carried out inspired by the framework of Spradley, and involved following the patients during visits to the department of oncology and in the psychiatric setting. Formal interviews were performed using semi-structured interview guides inspired by Kvale and Brinkmann. Patient files were examined focusing on the awareness of the psychiatric diagnosis and treatment and communication between the departments and sectors.

**Results:**

Our analysis showed one major theme: “Complexity on many levels”, and five subthemes: “The impact of the cancer trajectory on mental illness”, “The structure follows the disease, not the patient”, “Fragmentation of the health care system”, “Patient vulnerability” and “Importance of the patient-professional-relationship”. Barriers included lack of a systematic approach to the patient group in the health care system and sparse collaboration between departments and sectors. The cancer trajectory often led to severe worsening of the psychiatric illness, resulting in psychiatric hospitalisation. Facilitators were specialized coordinators at the hospital or municipality, relatives, patients’ resources and health professionals approaching the patient as a person rather than a disease. Final results will be ready for presentation at the conference.

**Conclusions:**

Despite intentions of reducing inequality, the Danish health care system is still not equipped to sufficiently help patients with cancer and pre-existing mental illness through their cancer treatment. This study will highlight relevant target points, paving the way for a new, feasible care model that improves continuity and patient-centered care for patients with cancer and mental illness.

**Disclosure of Interest:**

None Declared

